# Knockdown of CYP19A1 in Buffalo Follicular Granulosa Cells Results in Increased Progesterone Secretion and Promotes Cell Proliferation

**DOI:** 10.3389/fvets.2020.539496

**Published:** 2020-09-25

**Authors:** Xingrong Lu, Anqin Duan, Xiaoya Ma, Shasha Liang, Tingxian Deng

**Affiliations:** Guangxi Key Laboratory of Buffalo Genetics, Breeding and Reproduction Technology, Guangxi Buffalo Research Institute, Chinese Academy of Agricultural Science, Ministry of Agriculture, Nanning, China

**Keywords:** buffalo, CYP19A1, follicular granulosa cell, progesterone secretion, cell proliferation

## Abstract

Cytochrome P450 aromatase 19A1 (CYP19A1) is a critical enzyme in estrogen synthesis. However, the effect of CYP19A1 on cell growth and hormone secretion of buffalo follicular granulosa cells (BFGCs) is poorly understood. The objective of this study was to assess the role of CYP19A1 in cell proliferation and hormone secretion of BFGCs by knocking down CYP19A1 mRNA expression. The mRNA expression level of CYP19A1 gene was knocked down in BFGCs using the siCYP19A1-296 fragment with the best interference efficiency of 72.63%, as affirmed by real-time quantitative PCR (qPCR) and cell morphology analysis. The CYP19A1 knockdown promoted the proliferation of BFGCs through upregulating the mRNA expression levels of six proliferation-related genes (CCND1, CCNE1, CCNB1, CDK2, CDKN1A, and CDKN1B). Moreover, CYP19A1 knockdown increased (*P* < 0.05) the concentrations of progesterone secretion (P4) in BFGCs through increasing the mRNA expression levels of three steroidogenic genes (HSD17B1, HSD17B7, and CYP17A1). Our data further found that the FSH could inhibit the mRNA expression level of CYP19A1 in BFGCs, while LH obtains the opposite effect. These findings showed that the CYP19A1 knockdown had a regulatory role in the hormone secretion and cell proliferation in BFGCs.

## Introduction

With the action of reproductive hormones among the hypothalamic-pituitary-gonadal axis, follicular cells could promote the expression of steroid hormone synthase, guide the production of steroid hormones, and further regulate the growth and development of follicular. Estradiol (E2) produced by the interaction of steroid hormone synthase in follicular membrane cells and granulosa cells, which help in promoting the proliferation of granulosa cells and regulating the differentiation of follicular cells in the ovaries (1). In addition, E2 also promotes the sexual performance of female animals and physiological changes in the reproductive tract, the establishment of pregnancy, the development of breast catheters, calcium absorption (2).

Cholesterol side-chain cleavage enzyme is commonly referred to as P450SCC, which is a member of the cytochrome P450 family enzyme. The cytochrome P450 family 11 subfamily A member 1 (CYP11A1) converts cholesterol from follicular theca cells into progesterone (P5), which is the first and rate-limiting enzymatic step in the production of all steroid hormones (3). Subsequently, P5 is converted into progesterone (P4) by the action of 3beta-hydroxysteroid (HSD3B1) in follicular membrane cells, which is further transformed into androstenedione (A4) under the action of cytochrome P450 17A1 (CYP17A1) in granulosa cells (4). Notably, A4 is a precursor of estrone and estradiol that play a critical role in the estrogen synthesis (5, 6). A previous study has demonstrated that the Cytochrome P450 19A1 (CYP19A1, known as estrogen synthase) is a key enzyme that can convert A4 to Estradiol 1 (E1) during the estrogen synthesis (7). E1 could be converted into E2, by active of 17b-hydroxylase steroid dehydrogenase family, especially by the action of HSD17B1 (4). Alternatively, CYP19A1 may convert A4 to testosterone (8) that can further be transformed into E2 (9). Notably, numerous studies have reported that the E2 content tended to increase by increasing the mRNA expression levels of CYP19A1 and HSD17B1 in the bovine follicles grow from small to large initial stages (10–12). These results suggested that both CYP19A1 and HSD17B1 were the key enzymes in E2 production. However, limited information on the effects of HSD17B1 and CYP19A1 on the E2 secretion has been reported. Although the buffalo CYP19A1 has recently been successfully cloned and its tissue distribution analysis has been performed (13), the regulation function of CYP19A1 and its effect on the E2 secretion haven't been declared. Therefore, this study aims to investigate the effect of CYP19A1 on cell proliferation and hormone secretion in buffalo granulosa cells using RNA interference (RNAi) technology.

## Materials and Methods

### Cell Culture

A total of 24 ovaries from 12 Chinese local buffaloes were collected from a slaughterhouse in Guangxi Nanning, immediately stored in sterile 0.9%(w/v) NaCl at 37°C and transported to the laboratory within 4 h. The BFGCs were cultured and purified according to previously reported methods (14). Purified BFGCs were cultured in DMEM media supplemented with 10% FBS, penicillin (100 U·mL^−1^) and streptomycin (100 μg·mL^−1^) at 38.5°C in humidity and an atmosphere of 5% CO_2_ with a humidified incubator. Cells were used for subsequent experiments when the cell congruence reaches 90%. The remaining cells were stored immediately in liquid nitrogen.

### siRNA Experiments

A total of 3 siRNA fragments for buffalo CYP19A1 (GenBank accession number: KC020137.1) and control siRNA (NC) were recruited from the GenePharma Company (Shanghai, China). The sequences of siRNA fragments were listed in Supplementary Table 2. BFGCs were transfected with three CYP19A1 siRNA and NC at 50 nM concentration, using the Lipofectamine RNAiMAX Transfection Reagent (ThermoFisher, MA, USA) following the manufacturers' instructions. Total RNA were obtained from siRNA-transfected BFGCs after 72 h treatments. The siRNA transfection efficiency was monitored by qRT-PCR.

### RNA Isolation

Total RNA was isolated using the PureLink RNA Mini kit (Ambion, USA) following the manufacturer's instruction. RNA quantity and quality were evaluated using the NanoDrop ND-2000 spectrophotometer (NanoDrop Technologies, Wilmington, DE).

### Reverse Transcription and Quantitative Real-Time PCR

A total of 100 ng RNA for each sample was used for the preparation of cDNA by using the RevertAid First Strand cDNA Synthesis Kit (ThermoFisher Scientific, MA, USA). The qRT-PCR was conducted using the PowerUp SYBR Green Master Mix with the 20 μL reaction volume: PowerUp SYBR Green Master Mix 10 μL, RNase-free water 8 μL, upstream and downstream primers 0.5 μL, and cDNA 1 μL. The reaction procedure was run on the LightCycler 480 instrument (Roche, CH) under the following conditions: 95°C 30 s, 40 cycles of 95°C 5 s, 60°C 30 s. GAPDH was utilized for the reference gene, and the relative expression levels of the CYP19A1 gene in each experimental group were calculated by the 2^−ΔΔ*Ct*^ method (15). Primer pairs for all detected genes were shown in Supplementary Table 1.

### Cell Morphology Analysis

Changes in cell morphology of each experimental group were observed by microscopy after 72 h transfection, and the effects of each interference group on the growth morphology of BFGCs were compared.

### Cell Proliferation Assay

BFGCs were plated in 96-well culture plates and incubated in a growth medium complemented with 10% FBS until 60% confluence. Cell proliferation was evaluated after 72 h transfection, using the CCK-8 cell proliferation Kit (KeyGEN BioTECH, Jiangsu, China) according to the manufacturer's instruction. Add cell proliferation detection fluid with 10 μL per cell hole, incubated for 4 h at 37°C. Overall, the proliferation for each optimal interference group was detected at the 24, 48, and 72 h, respectively. The cell activity is expressed by optical density (OD) value in 450 nm wavelengths.

### Cell Apoptosis Assay

Annexin V-EGFP Kit (KeyGEN BioTECH, Jiangsu, China) was selected for the cell apoptosis analysis according to the manufacturer's instruction. Briefly, the cells were firstly treated with the pancreatic enzymes without EDTA after the CYP19A1 knockdown for 72 h, followed by washing two times with PBS, suspending cells with 500 μL binding Buffer, blending the cells with the 5 μL annexin V-EGFP Mix and 5 μL propidium iodide. After the mixed reaction was in room temperature and light avoidance of 10–20 min, BFGCs were observed and detected under the fluorescence microscope. For them, green light cells indicate early apoptosis cells, and red-light cells indicate apoptosis in the mid and late-stage and necrotic cells. Effects of CYP19A1 knockdown on apoptosis in experimental groups were analyzed and compared, and the calculation formula was as follows: the rate of apoptosis cells = number of apoptosis cells/numbers of total cells, the rate of living cell = number of live cells/numbers of total cells.

### Enzyme-Linked Immunosorbent Assay (ELISA)

The levels of E2 and P4 were determined in the cell culture supernatants using ELISA kit (Beijing Kangpu Tongchuang Biotechnology Co., Ltd, Beijing, China) after 72 h transfection. In this study, three repeating experiments were designed, each of which was repeated three times. The results were calculated based on standard density: 120, 80, 40, 20, 10 ng/L.

### Statistical Analysis

The data were expressed as mean ± SEM. Tukey *post hoc* test and one-way ANONA analysis were used to determine the statistical significance. Each test was conducted in triplicate. Data analysis was performed by SPSS 16.0 software (SPSS Inc., Chicago, IL, USA) and GraphPad Prism 7.0 (GraphPad Prism Software Inc., San Diego, USA).

## Results

### siRNA Interference Efficiency Detection of CYP19A1

**Figure 1:**
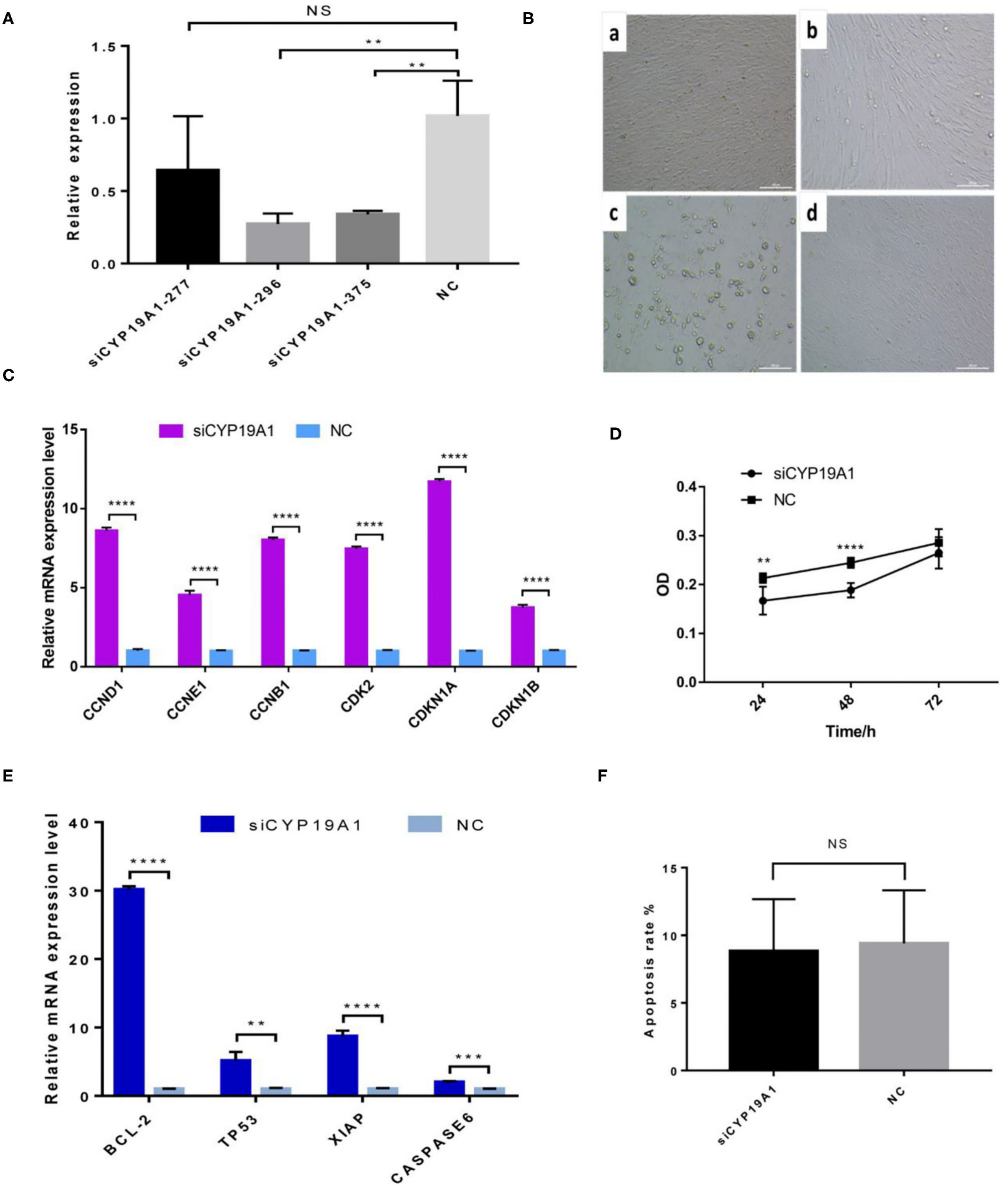
CYP19A1 induced cell proliferation and apoptosis of BFGCs. **(A)** A screen of CYP19A1 siRNA fragments in BFGCs. **(B)** Changes of cell morphology after CYP19A1 interference for 72 h. 200 × (bar = 100 μm). Note a: BFGCs transfected with siCYP19A1-277 for 72 h; b: BFGCs transfected with siCYP19A1-296 for 72 h; c: BFGCs transfected with siCYP19A1-375 for 72 h; d: BFGCs transfected with NC for 72 h. **(C)** Effect of CYP19A1 gene interference on proliferation-related genes expression. **(D)** Effect of siCYP19A1 on BFGCs by CCK-8 cck8 detection. **(E)** Effect of CYP19A1 gene interference on apoptosis-related genes expression. **(F)** Comparison of apoptosis rates between test group and native control group. **P < 0.01; ***P < 0.001; ****P < 0.0001; NS, no significant.

The interference efficiency of three siRNA fragments of CYP19A1 was verified by BFGCs transfection. As shown in Figure 1A and Supplementary Table 3, all the siRNA fragments had a inhibitory effect on the CYP19A1 of BFGC, and siCYP19A1-296 showed the best interference efficiency (72.63%, *P* = 0.007) compared to that of siCYP19A1-277 (35.86%, *P* = 0.217) and siCYP19A1-375 (66.03%, *P* = 0.0086). Moreover, the BFGCs treated by siCYP19A1-296 remained a flat elongated shape after 72 h, with a small number of cells began to enter the early apoptosis (Figure 1B). These results indicated that the siCYP19A1-296 can serve as the best siRNA fragments and used for further analysis.

### CYP19A1 Knockdown Altered BFGCs Proliferation

The effect of CYP19A1 knockdown on cell proliferation was investigated at three time-points (24, 48, and 72 h). Compared with the NC group, BFGCs treated by the CYP19A1 knockdown increased cell population (*P* < 0.05) in the culture medium (Figure 1D and Supplementary Table 4). Subsequently, mRNA levels of six proliferation-related genes (CCND1, CCNE1, CCNB1, CDK2, CDKN1A, and CDKN1B) were further determined after CYP19A1 knockdown using qPCR. The results indicated that the increases (*P* < 0.05) in the mRNA expression level of the studied genes were observed after CYP19A1 silencing (Figure 1C). These findings indicate that CYP19A1 plays a key role in BFGC growth.

### Effects of CYP19A1 Knockdown on BFGC Apoptosis

To determine apoptosis regulation of CYP19A1 in BFGC, apoptosis cells were detected by Annexin V-EGFP Kit. Figure 1E showed no effect on the apoptosis rate (*P* > 0.05) between them (Figure 1F). Interestingly, when the mRNA expression levels of apoptosis inducers were quantified, we found that the CYP19A1 knockdown increased (*P* < 0.05) the expression of the apoptosis genes (TP53 and CASPASE6) and anti-apoptosis genes (Bcl-2 and XIAP), and the latter had higher (*P* < 0.05) expression level compared to that of the former.

### CYP19A1 Knockdown Increased the Concentrations of Progesterone in BFGC

**Figure 2:**
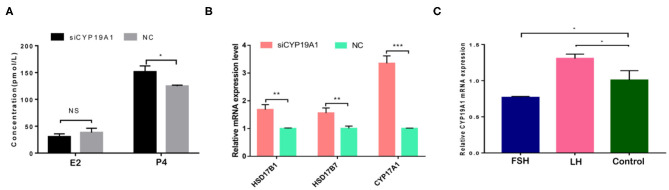
CYP19A1 regulated hormone secretion of BFGCs. **(A)** Effects of E2 and P4 secretion from BFGCs with CYP19A1 interference; **(B)** Effect of CYP19A1 knockdown on steroidogenic gene expression. **(C)** Effects of adding LH and FSH on CYP19A1 expression. *P < 0.05; **P < 0.01; ***P < 0.001.

To verify the effect of silencing CYP19A1 on steroid hormone levels, the concentrations of E2 and P4 in the culture medium at 48 h post-transfection were observed. Results showed that P4 levels in the CYP19A1 knockdown group were higher (*P* < 0.05) than that of the NC group after transfection. There was no difference between the E2 group and NC (Figure 2A; Table 1). The mRNA expression of steroidogenic genes, including HSD17B1, HSD17B7, and CYP17A1 was further analyzed. The results showed that CYP19A1 knockdown increased (*P* < 0.05) in HSD17B1, HSD17B7, and CYP17A1 at the mRNA expression level (Figure 2B). Moreover, we also determined the mRNA expression of several upstream genes (FSH and LH). The results showed that the FSH decreased (*P* < 0.05) the expression of the CYP19A1. Whereas, the LH increased (*P* < 0.05) the CYP19A1 gene expression (Figure 2C). These results confirmed that P4 secretion was controlled by CYP19A1 in BFGCs.

**Table 1:** Effects of E2 and P4 secretion from BFGCs with CYP19A1 interference.

**Groups (*n* = 3)**	**Concentration of E2 (pmol/L) (Mean ± SD)**	**Concentration of P4 (pmol/L) (Mean ± SD)**
siCYP19A1	30.35 ± 3.20	151.46 ± 6.32^a^
NC	38.22 ± 4.63	124.81 ± 1.06^b^

a, b *Values within each column are significantly different (p < 0.05)*.

## Discussion

Evidence indicated that the growth and development of follicles are mediated by hormones and growth factors secreted from granulosa cells (16), including E2 and P4 (17, 18). Previous studies have demonstrated that both E2 and P4 play a vital role in the steroid hormone synthesis involved in the regulation of granulosa cells proliferation and differentiation in the ovaries (1, 19, 20). CYP19A1 is a critical enzyme in the E2 synthesis, however, there are limited reports about the molecular mechanism of CYP19A1 regulating BFGCs proliferation, apoptosis, and hormone synthesis. To explore these issues, we designed and synthesized the interference fragment, siCYP19A1, to silence CYP19A1 mRNA expression in BFGCs. Then, we investigated the effects of CYP19A1 knockdown on cell proliferation, apoptosis, and hormone production of BFGCs. In the present study, we found that there was a strong inhibitory effect on the proliferation of BFGCs after CYP19A1 knockdown for 24 and 48 h, and this inhibition would decrease as the cells continued to divide after 72 h. This finding suggested that the CYP19A1 gene could be considered as candidate genes affecting the BFGC growth.

To gain insight into the mechanisms that CYP19A1 prevents the BFGC growth, we assessed the changes in mRNA expression of key proliferation-related genes (CCND1, CCNE1, CCNB1, CDK2, CDKN1A, and CDKN1B). Our data showed all the proliferation-related genes appeared to be up-regulated after the CYP19A1 knockdown. Previous studies have demonstrated that all of them play important regulatory roles in cell proliferation and the cell cycle (21–24). For example (25), found that neuron growth factors (NGF) promote the proliferation of granulosa cells (GC) by down-regulating ESR2 and CDKN1A. A similar result was also supported by our data. These results showed that CYP19A1 was an essential gene to maintain the growth and proliferation of BFGCs.

Apoptosis is a cellular mechanism of ovarian follicular atresia and a physiological form of cell death. Previous studies have demonstrated that Bcl-2 gene family members regulate the apoptosis of granulosa cells (26, 27). As the CYP19A1 knockdown has been shown to impact on the cell proliferation, we hypothesized that the knockdown of CYP19A1 may promote cell survival by reducing apoptosis rate of BFGCs. However, we determined that the knockdown of CYP19A1 had no effect on the apoptosis of BFGCs. At the mRNA level, we observed the mRNA expression level of anti-apoptosis genes (Bcl-2 and XIAP) was higher (*P* < 0.05) than apoptosis genes (TP53 and CASPASE6) after CYP19A1 knockdown. Taken together, we speculated that the knockdown of CYP19A1 may affect the mRNA expression level of various apoptosis-related factors, but there is no effect on apoptosis rates in BFGC.

Granulosa cells are key cells in hormone secretion, and CYP19A1 plays an important role in hormone circulation in the body (4, 28). In this study, steroid hormone secretion detection analysis revealed that the CYP19A1 knockdown increased the secretion of P4 without changing the secretion of E2 in BFGCs. Meanwhile, our data showed an increase (*P* < 0.05) in expression of steroid-related gene HSD17B1, HSD17B7, and CYP17A1after CYP19A1 knockdown, accompanied by an increase in P4 secretion. However, the reasons for CYP19A1 knockdown not causing the change of E2 secretion have yet to be further verified, maybe HSD17B1 and HSD17B7 were antagonistic in regulating E1 to E2 conversions, there was a regulatory link that has not been confirmed.

**Figure 3:**
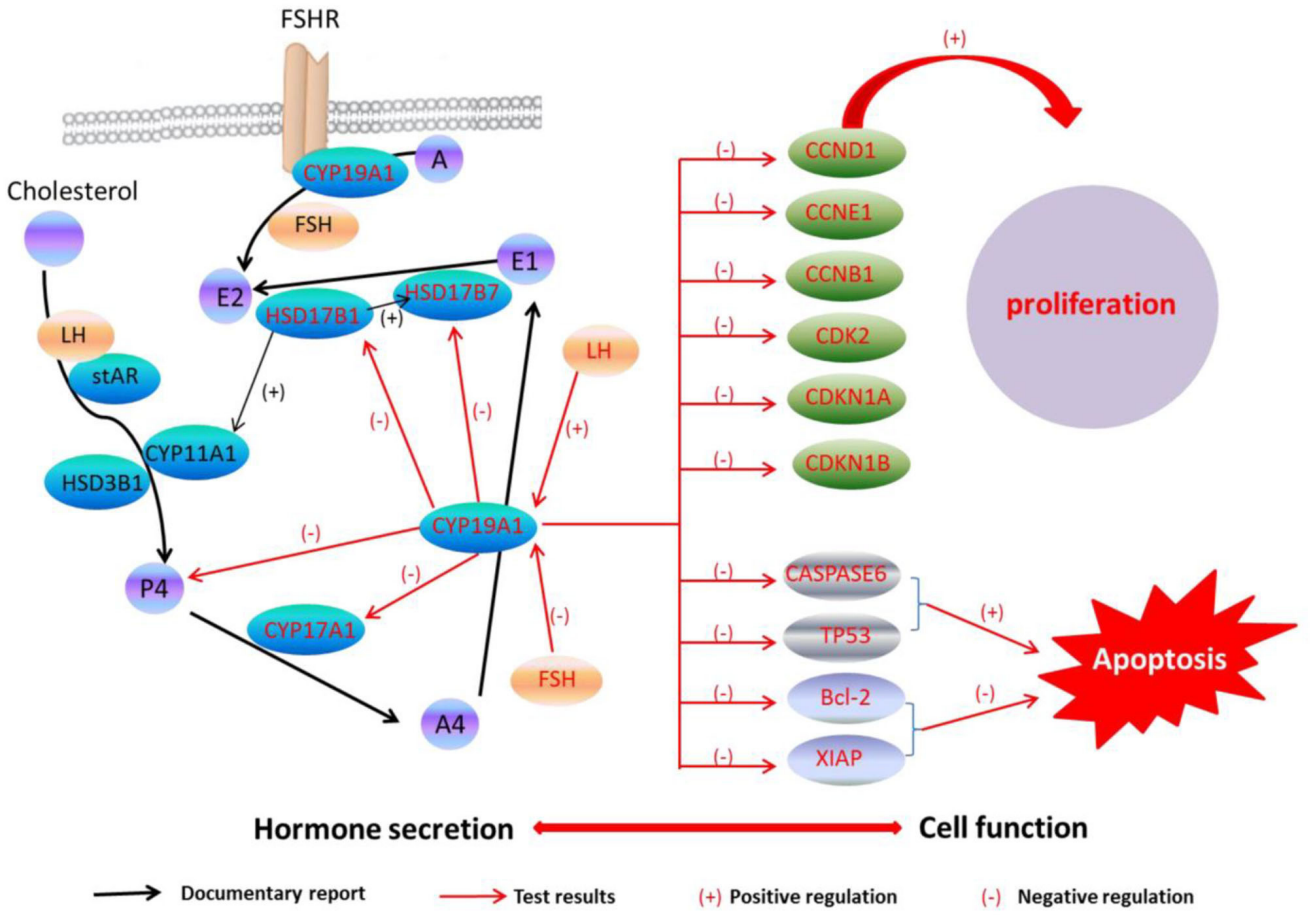
CYP19A1 regulated cell function and hormone secretion of BFGCs. Red font represents test results.

A previous study has found that the FSH adding may raise the E2 and P4 levels during the granulosa cells culture *in vitro*, while the secreting level of E2 and P4 was increased by adding the LH in a small quantity (29). Because of CYP19A1 is a key enzyme which involves in E2 synthesis, the expression of CYP19A1 was regulated by FSH and LH in the mRNA level. Our data further showed that FSH adding to the medium reduced the relative expression of CYP19A1 in BFGCs, whereas adding LH could increase the relative expression of CYP19A1 (Figure 3). It can be inferred that CYP19A1 plays an important role in maintaining the stability of hormone secretion in the body.

## Conclusion

In this work, we confirmed that the CYP19A1 knockdown may promote BFGCs cell proliferation through regulating the proliferation-related gene expressions. We also confirmed that the CYP19A1 knockdown may increase P4 synthesis by controlling the expression of steroidogenic genes. Our findings provided insight to reveal the relationship between the structural function of granulosa cells and hormone secretion.

## Data Availability Statement

All datasets generated for this study are included in the article/Supplementary Material.

## Ethics Statement

This study has been approved by the Ethics Committee of Buffalo Research Institute, Chinese Academy of Agricultural Sciences (BRI-CAAS), Nanning, China (approval code GXBRI-200701).

## Author Contributions

TD and XL were in charge of designing experiments and analyzing data. XL and AD were responsible for preparing the initial draft of the manuscript. XL and TD were in charge of approving the final draft of the manuscript and were responsible for cell culture, cell proliferation, apoptosis assays, and data collection. XM and SL were responsible for qPCR analysis and enzyme assay testing and data collection. All the authors have examined the manuscript.

## Conflict of Interest

The authors declare that the research was conducted in the absence of any commercial or financial relationships that could be construed as a potential conflict of interest.
