# Supplementary figures and images for: Knockdown of CYP19A1 in Buffalo Follicular Granulosa Cells Results in Increased Progesterone Secretion and Promotes Cell Proliferation
Source: Front Vet Sci. 2020 Sep 25;7:539496. doi: 10.3389/fvets.2020.539496 (PMC7545956; doi:10.3389/fvets.2020.539496)

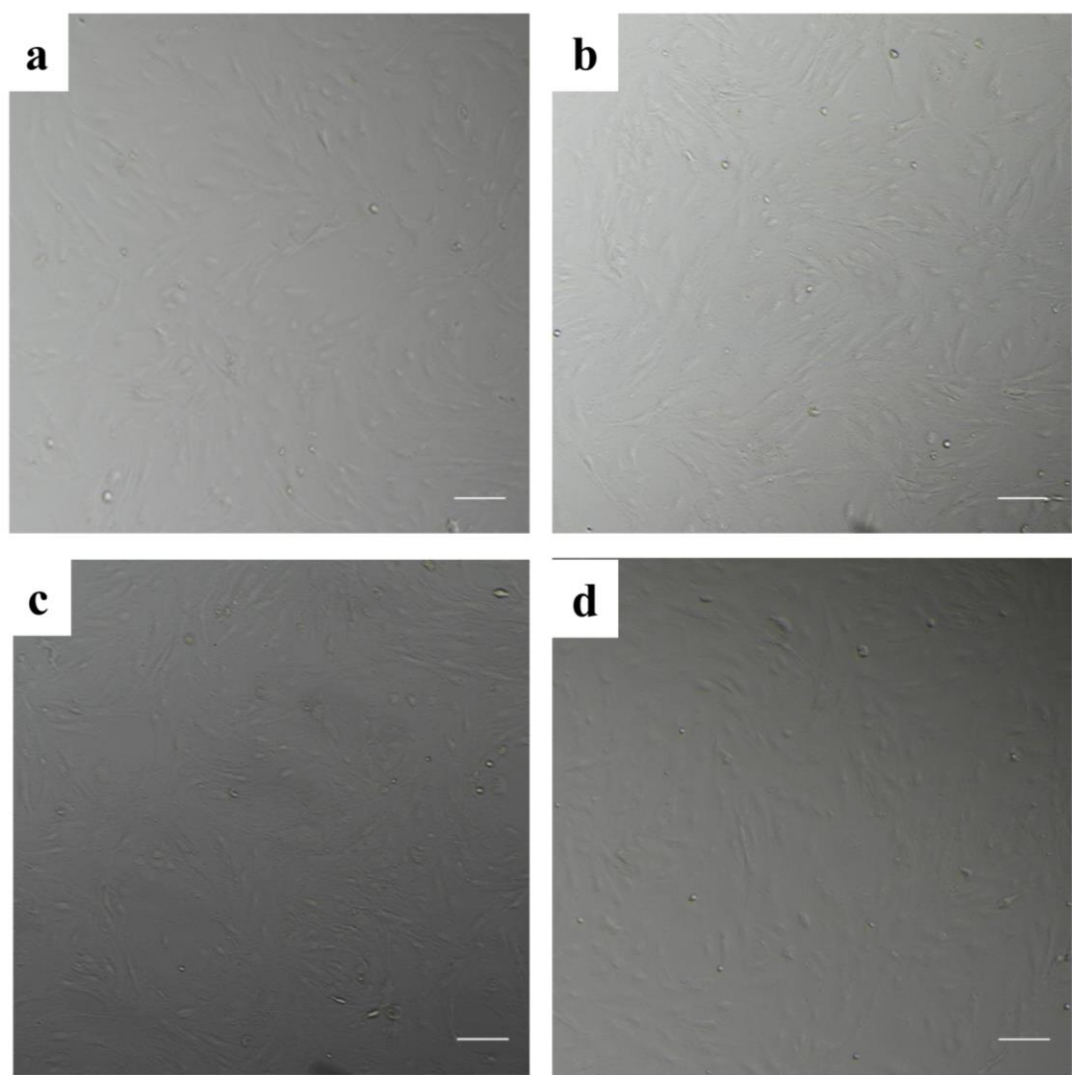

**Figure S1. Vectors transfection in to BFGC**

Supplement: Supplementary file 5 [file Data_Sheet_1.PDF]
